# Successful Management of COVID-19-Associated Systemic Capillary Leak Syndrome Using Veno-Venous Extracorporeal Membrane Oxygenation: A Case Report

**DOI:** 10.7759/cureus.76657

**Published:** 2024-12-31

**Authors:** Misa Kitamura, Hiromu Okano, Satoshi Jujo, Kenji Ishii, Kazuhiro Aoki, Hiroshi Okamoto

**Affiliations:** 1 Department of Internal Medicine, St. Luke's International Hospital, Tokyo, JPN; 2 Department of Social Medical Sciences, Graduate School of Medicine, International University of Health and Welfare, Tokyo, JPN; 3 Department of Critical Care Medicine, St. Luke's International Hospital, Tokyo, JPN

**Keywords:** covid-19, covid-19-associated scls, extracorporeal membrane oxygenation support, systemic capillary leak syndrome, v-v ecmo

## Abstract

Systemic capillary leak syndrome (SCLS) is a rare and life-threatening disorder characterized by acute hypotension, hypoalbuminemia, and hemoconcentration, which often results in severe respiratory complications, such as pulmonary edema. SCLS can be triggered by infections, including COVID-19, and is associated with a high mortality rate. Here, we report a case of COVID-19-associated SCLS in a 68-year-old man. After aggressive fluid resuscitation, the patient’s respiratory failure worsened. Veno-venous extracorporeal membrane oxygenation (V-V ECMO) was initiated early because of rapidly declining oxygenation (PaO_2_/FiO_2_=58) and pulmonary compromise. V-V ECMO successfully supported respiratory and circulatory functions and stabilized the patient. The patient was successfully weaned from ECMO on day 10 and later discharged from the ICU in stable condition. This case highlights the potential benefits of early ECMO intervention and controlled fluid resuscitation in managing COVID-19-associated SCLS.

## Introduction

Systemic capillary leak syndrome (SCLS) is a rare and potentially fatal condition, with a mean age of onset around 45 years. Pediatric and geriatric cases are rare [1]. It is characterized by acute hypotension, hypoalbuminemia, and hemoconcentration and was first described by Clarkson in 1960 [2]. Its exact pathophysiology remains unclear; however, it is thought to involve increased vascular permeability caused by endothelial dysfunction and leads to the rapid extravasation of plasma components [3].

This condition typically necessitates large-volume fluid resuscitation, which can result in severe respiratory distress owing to pulmonary edema and cause challenges in respiratory management [4]. Herein, we report a case of COVID-19-associated SCLS that was successfully treated with early veno-venous extracorporeal membrane oxygenation (V-V ECMO), resulting in a favorable outcome.

## Case presentation

A 68-year-old man with a 40-pack-year smoking history presented with fever and cough; he had received six doses of the COVID-19 vaccine. The patient tested positive for COVID-19 and was admitted to the hospital due to altered mental status. On admission, the following were noted: the blood pressure: 115/88 mmHg, heart rate: 115 beats/min, respiratory rate: 18 breaths/min, and oxygen saturation: 94% on room air. Physical examination results were unremarkable. Laboratory findings revealed hypoalbuminemia (1.7 g/dL), elevated hemoglobin (22.1 g/dL), hematocrit (62%), and lactate levels (4.5 mmol/L) on admission. After fluid resuscitation, by ICU day 2, albumin levels improved to 2.7 g/dL, hemoglobin levels decreased to 16.8 g/dL, and hematocrit levels reduced to 47% (Table 1). Transthoracic echocardiography revealed a preserved left ventricular ejection fraction (LVEF) (60%) with a collapsed inferior vena cava. Chest computed tomography (CT) revealed granular shadows in both the upper lobes.

**Table 1:** Blood test results at the time of admission, showing signs of hemoconcentration, hypoalbuminemia, and hyperlactatemia

Test Item	Result	Unit	Reference range
White blood cell (WBC) count	23.5	10^3^/μL	3.3-8.6
Red blood cell (RBC) count	7.27	10^6^/μL	-
Hemoglobin (Hb)	22.1	g/dL	13.7-16.8
Hematocrit	62	%	40.7-50.1
Platelet count	18.4	10^4^/μL	15.8-34.8
Albumin	1.7	g/dL	4.1-5.1
Blood urea nitrogen (BUN)	24.4	mg/dL	8-20
Creatinine	0.6	mg/dL	0.65-1.07
Aspartate aminotransferase (AST)	11	U/L	-
Alanine transaminase (ALT)	7	U/L	10-42
Sodium (Na)	136	mEq/L	138-145
Potassium (K)	4.3	mEq/L	3.6-4.8
Chloride (Cl)	101	mEq/L	-
C-reactive protein (CRP)	1.65	mg/dL	-0.14
Procalcitonin	0.16	ng/mL	-0.05
pH	7.423	-	7.35-7.45
pO_2_	87.5	mmHg	80-100
pCO_2_	26.8	mmHg	35-45
Lactate	4.5	mmol/L	0.36-0.75
SARS-CoV-2 antigen	Positive	-	-

The patient was diagnosed with COVID-19 and treated with remdesivir (200 mg on day 1 and 100 mg on days 2-9). Based on the hypoalbuminemia and elevated hematocrit levels, SCLS was suspected. However, heart failure and septic shock were also considered differential diagnoses and fluid resuscitation with balanced crystalloid solutions was initiated to address these possibilities.

Notably, seven hours after admission, the patient developed rapid atrial fibrillation with a pulse rate of 170 bpm and thereafter exhibited hypotension and agitation. The patient underwent successful electrical cardioversion with a return to sinus rhythm; however, without the administration of 5% albumin and balanced crystalloids, his pulse rate increased to 120-130 bpm, resulting in recurrent hypotension. Subsequently, intravenous norepinephrine and vasopressin were initiated. A total volume of approximately 10 L of balanced crystalloid fluids was administered within 24 hours of admission.

**Figure 1:**
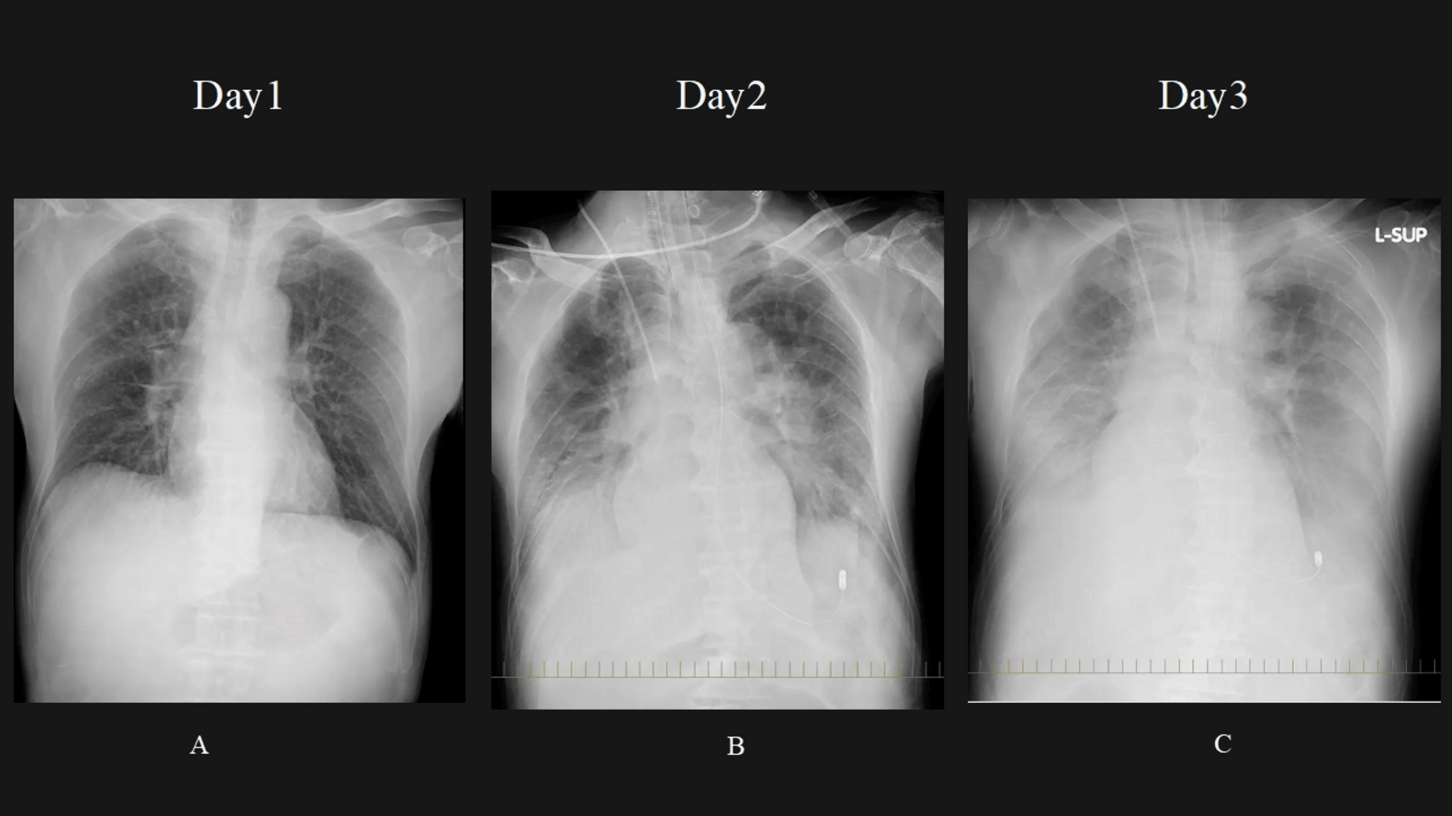
Changes in chest X-ray since admission Day 1 (A): The initial chest X-ray shows the baseline condition of the lung fields. Day 2 (B): Gradual worsening of lung field infiltration shadow is observed. This is partly attributed to volume loading, which may have caused an increase in the density or the extent of the shadow in the lungs. Day 3 (C): Further progression of lung field infiltration is noted, with continued worsening from the previous days. The volume loading effect may still be contributing to this change.

Respiratory status rapidly worsened to an arterial oxygen partial pressure to inspired oxygen fraction (P/F) ratio of 85, and his respiratory rate increased to approximately 40 breaths/min. Given this rapid deterioration, we performed endotracheal intubation within six hours of admission, in the early hours of day 2. Chest radiography showed progressive infiltrative changes in both lung fields, which was partly attributed to volume overload (Figure 1). Thoracic drainage via chest tube insertion was considered; however, due to hemodynamic instability and concerns about potential blood pressure reduction following drain placement, the chest tube insertion was not performed. The P/F ratio was 58, with 75% lung opacities on the chest radiograph, positive end-expiratory pressure of 8 cm H₂O, lung compliance of 45 mL/cm H₂O, and a Murray score [5] of 10. Based on these findings, V-V ECMO was initiated on day 3. Although SCLS was suspected, the initial presentation required standard resuscitation protocols to rule out other critical conditions such as septic shock, making immediate ECMO initiation impractical without clear diagnostic and respiratory criteria.

**Figure 2:**
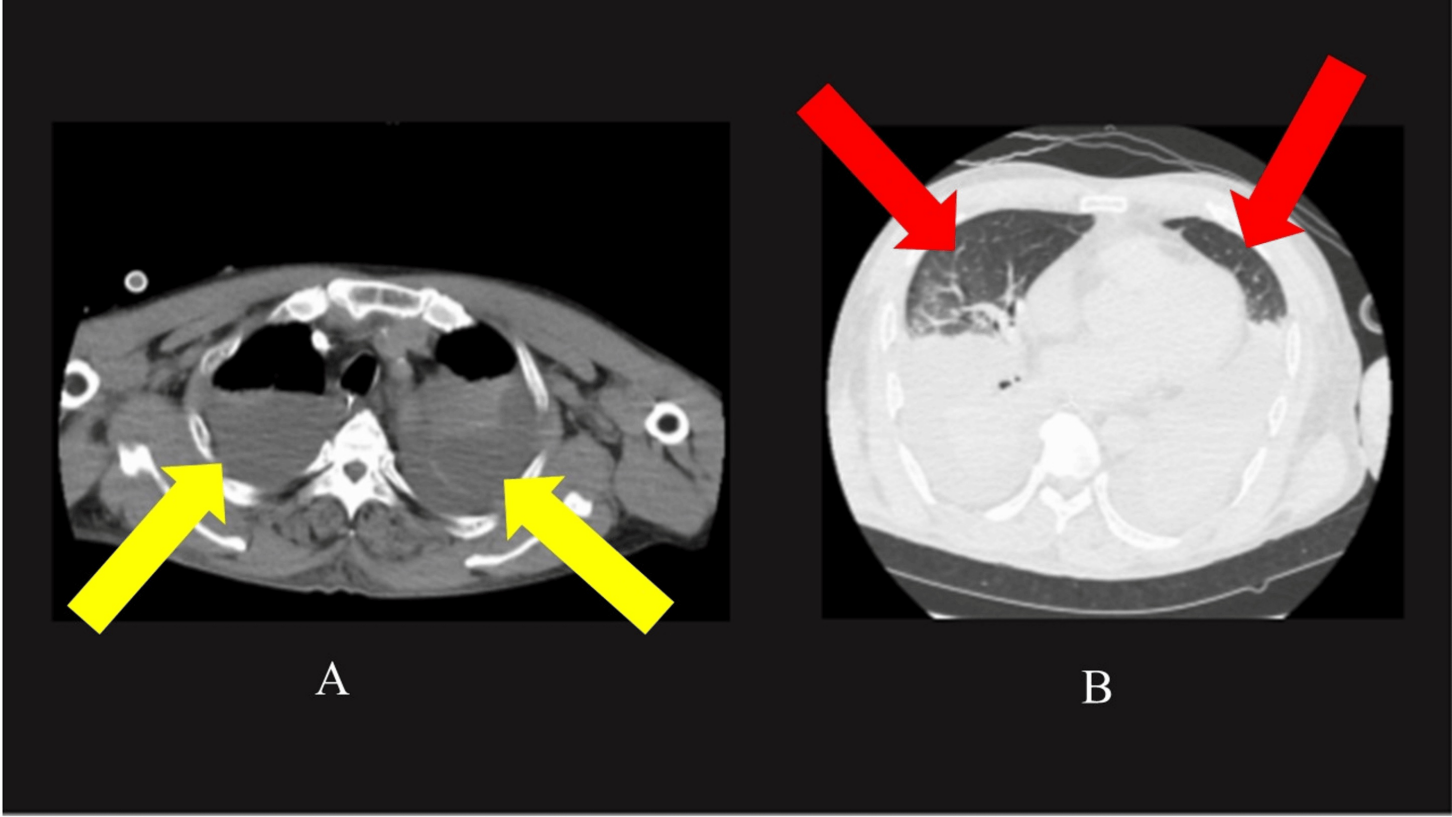
Plain CT of the lung taken after the introduction of ECMO A: In the mediastinal window, bilateral pleural effusion and atelectasis (yellow arrows) are observed. B: In the lung window, only a small portion of normal lung tissue is visible (red arrow). ECMO, extracorporeal membrane oxygenation; CT, computed tomography

**Figure 3:**
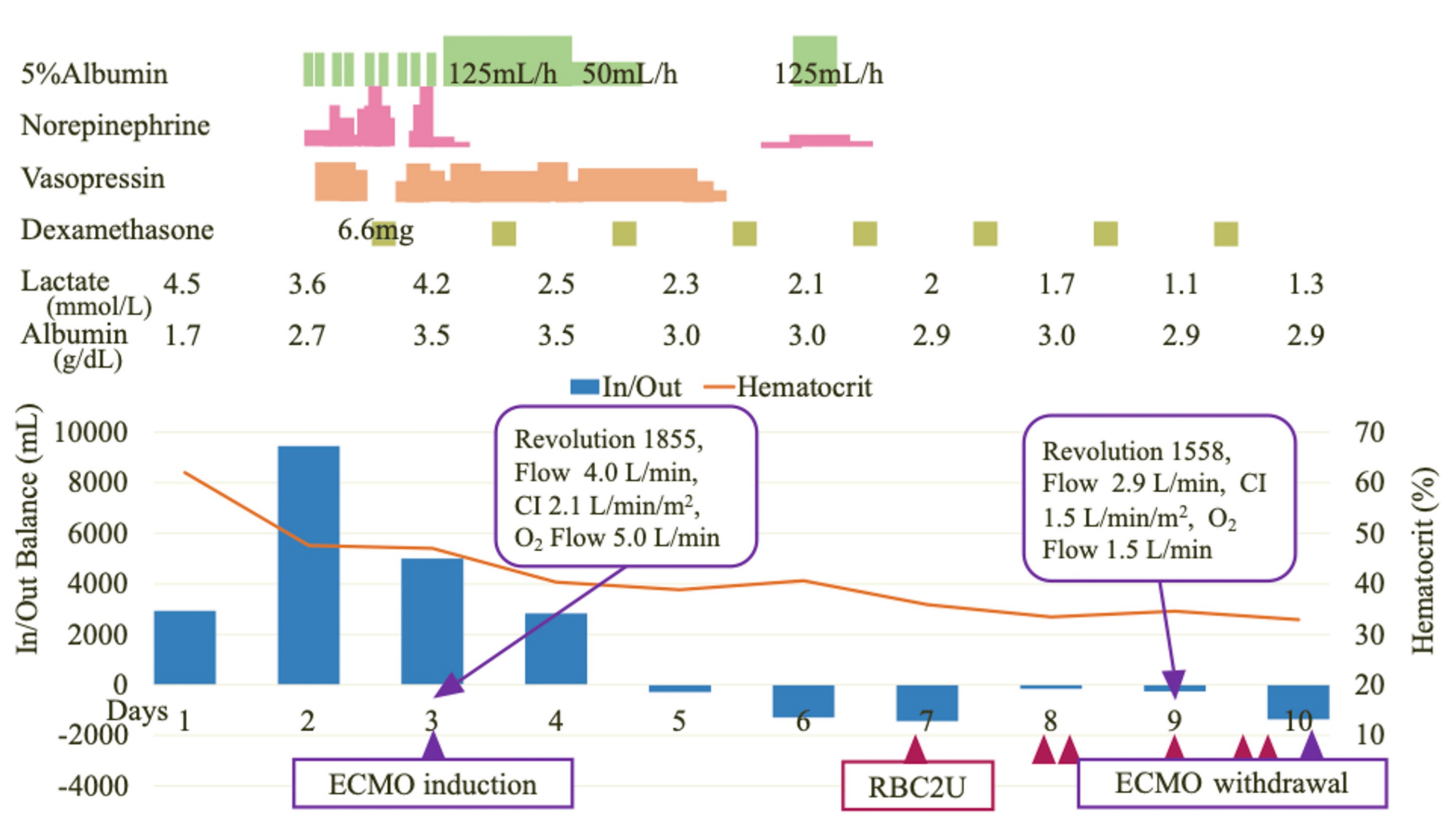
The clinical course of this case V-V ECMO was initiated on day 3. Albumin administration (green bar), norepinephrine (pink bar), and vasopressin (orange bar) were continuously administered, with gradual tapering and eventual cessation over time. The overall balance began to turn negative on day 5 (blue bar), and ECMO was successfully weaned on day 10. Daily albumin and lactate values are shown to demonstrate the response to fluid management. CI, Cardiac Index; V-V ECMO, veno-venous extracorporeal membrane oxygenation

**Figure 4:**
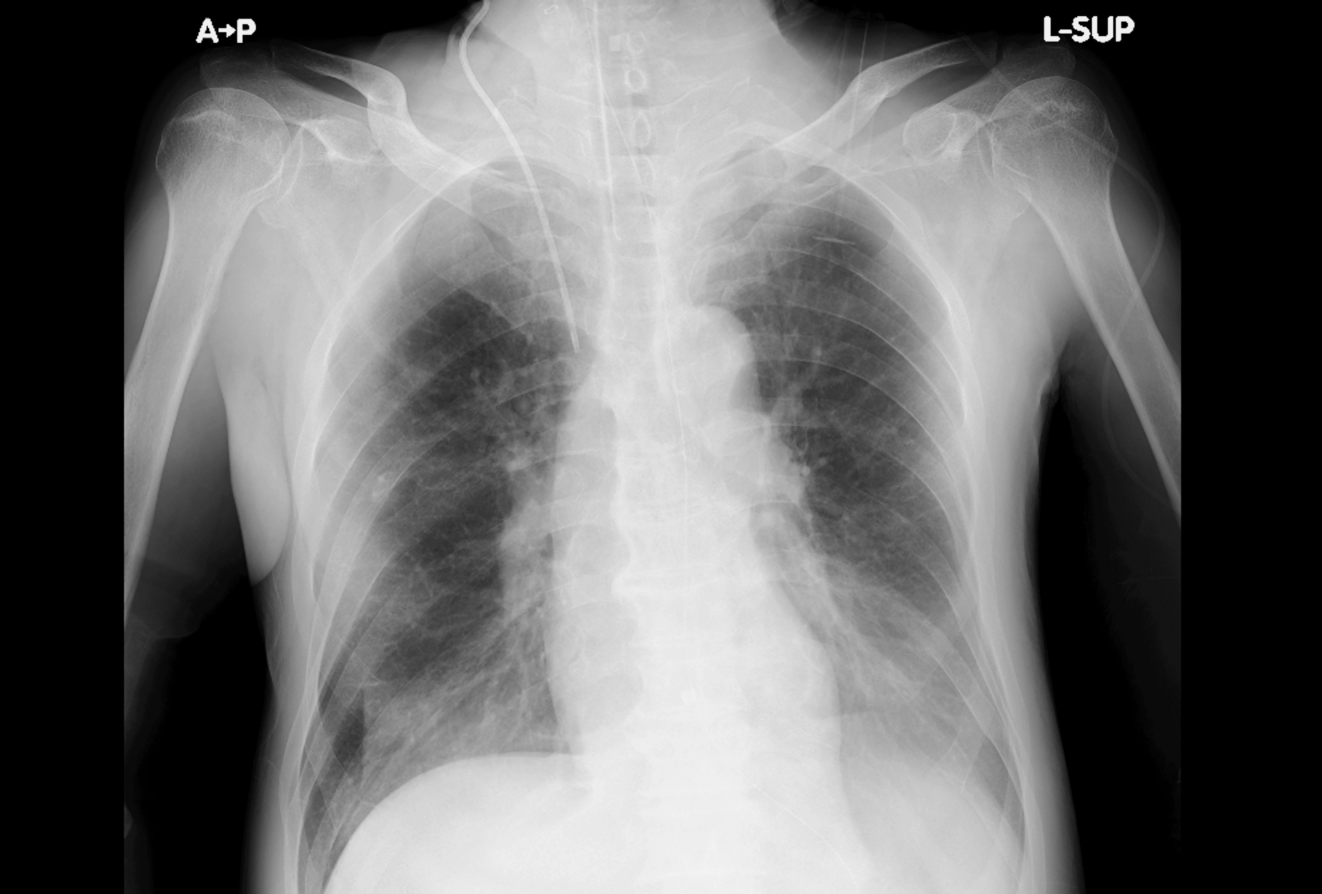
Chest X-ray on day 25 The chest X-ray on day 25 also showed improvement.

Chest CT performed after the initiation of V-V ECMO showed significant bilateral pleural effusion and atelectasis (Figure 2). We continued to administer 5% albumin to maintain the extracorporeal membrane oxygenation (ECMO) flow at least 3 L/min. We discontinued continuous albumin administration on day 5, and the norepinephrine demand ceased on the same day. The overall fluid valence became negative. With sustained negative fluid balance confirmed daily, the ECMO flow was gradually lowered and removed on day 10 (Figure 3). The patient underwent tracheostomy on day 18, was successfully liberated from mechanical ventilation on day 20, and the chest X-ray on day 25 also showed improvement, with oxygen therapy discontinued (Figure 4). The patient was discharged from the ICU on day 31.

## Discussion

In this case, a diagnosis of SCLS was made based on the recovery of hemoconcentration and hemodynamics without specific treatment and the detection of IgG-type M protein in blood tests on ICU day 18. Treatment with V-V ECMO was chosen instead of inserting a chest drain due to concerns about hemodynamic stability. It is also suggested that, in the absence of such hemodynamic concerns, improvement might have been achieved without the need for V-V ECMO.

SCLS is a severe and life-threatening condition. It can be triggered by mild infections, such as upper respiratory tract infections, and other factors, including vaccinations and hematologic malignancies [6]. To date, only 14 cases of SCLS associated with COVID-19 infection or vaccination-related SCLS have been reported [4,7]. Of the reported cases, seven patients died, resulting in a mortality rate of 50% [4,7], which was significantly higher than the 30% mortality reported for non-COVID-19-associated SCLS [8]. Despite the high mortality rate, the clinical course of COVID-19-associated SCLS remains poorly understood because of the limited number of cases. This case report provides valuable clinical insight into the management of COVID-19-associated SCLS, particularly emphasizing the importance of early recognition of the condition and careful hemodynamic management. Our findings suggest that monitoring hemoconcentration and hemodynamics, combined with timely intervention to stabilize these parameters, may be critical in improving outcomes.

This represents the first reported case of a patient requiring V-V ECMO for COVID-19-associated SCLS. In SCLS, only two cases of Veno-arterial extracorporeal membrane oxygenation (VA-ECMO) use have been reported [9,10]. In both cases, cardiac function was impaired due to myocardial edema and was supported by V-A ECMO, leading to patient survival. The indications for V-A ECMO have not been established [11]. However, improved survival rates have been reported with V-A ECMO for severe sepsis-induced cardiogenic shock, in which all patients had severe myocardial dysfunction with an LVEF of ≤35% [12]. Moreover, a systematic review revealed that V-A ECMO increased survival rates in patients with septic shock with compromised cardiac function [13]. They reported that survival among patients with LVEF <20% was significantly higher than those with LVEF >35%. In this case, we chose V-V ECMO rather than V-A ECMO because of a preserved LVEF of 57%. If the cardiac function is preserved, V-V ECMO can ensure oxygenation, enabling the stabilization of both respiratory and circulatory functions.

ECMO might serve as a therapeutic option in treating SCLS. SCLS requires early and aggressive external fluid administration for shock; however, pulmonary edema is a concerning complication. The peak of shock in SCLS is reported to be at 24-36 hours [14], suggesting that an appropriately timed initiation of ECMO may help overcome the peak of shock and improve patient outcomes. In this case, ECMO initiation at the appropriate time and pertinent fluid management may have contributed to the patient’s survival.

The key to successful ECMO is avoiding excessive fluid infusion. Although adequate fluid resuscitation is crucial initially, excessive fluid administration leading to fluid overload can contribute to prolonged and worsening multiple organ failure. In the 2021 Surviving Sepsis Guidelines, using albumin is considered an option to avoid large volumes of crystalloids [15]. Albumin fluid resuscitation in patients who have undergone V-A ECMO has been associated with improved survival outcomes [16]. However, there is no consensus on the optimal albumin concentration [15]. Therefore, avoiding excessive fluid infusion and preventing the worsening of organ dysfunction may have contributed to the improvement in favorable outcomes. 

## Conclusions

This report demonstrated a successful case of V-V ECMO in a patient with COVID-19-associated SCLS. Early initiation of ECMO played a crucial role in stabilizing respiratory and circulatory functions. Careful fluid management, including initial aggressive resuscitation followed by avoiding fluid overload, contributed to a favorable outcome. This case highlights the importance of timely ECMO initiation and meticulous fluid management in the treatment of COVID-19-associated SCLS.
